# Dopamine therapy does not affect cerebral autoregulation during hypotension in newborn piglets

**DOI:** 10.1371/journal.pone.0170738

**Published:** 2017-01-31

**Authors:** Vibeke Ramsgaard Eriksen, Martin Bo Rasmussen, Gitte Holst Hahn, Gorm Greisen

**Affiliations:** 1 Department of Neonatology, Copenhagen University Hospital – Rigshospitalet, Copenhagen, Denmark; 2 University of Copenhagen, Faculty of Health and Medical Sciences, Copenhagen, Denmark; 3 Department of Pediatrics, Copenhagen University Hospital – Hvidovre Hospital, Hvidovre, Denmark; University of Southampton, UNITED KINGDOM

## Abstract

**Background:**

Hypotensive neonates who have been treated with dopamine have poorer neurodevelopmental outcome than those who have not been treated with dopamine. We speculate that dopamine stimulates adrenoceptors on cerebral arteries causing cerebral vasoconstriction. This vasoconstriction might lead to a rightward shift of the cerebral autoregulatory curve; consequently, infants treated with dopamine would have a higher risk of low cerebral blood flow at a blood pressure that is otherwise considered “safe”.

**Methods:**

In anaesthetized piglets, perfusion of the brain, monitored with laser-doppler flowmetry, and cerebral venous saturation was measured at different levels of hypotension. Each piglet was studied in two phases: a phase with stepwise decreases in MAP and a phase with stepwise increases in MAP. We randomized the order of the two phases, whether dopamine was given in the first or second phase, and the infusion rate of dopamine (10, 25, or 40 μg/kg/min). In/deflation of a balloon catheter, placed in vena cava, induced different levels of hypotension. At each level of hypotension, fluctuations in MAP were induced by in/deflations of a balloon catheter in descending aorta.

**Results:**

During measurements, PaCO_2_ and arterial saturation were stable. MAP levels ranged between 14 and 82 mmHg. Cerebral autoregulation (CA) capacity was calculated as the ratio between %-change in cerebrovascular resistance and %-change in MAP induced by the in/deflation of the arterial balloon. A breakpoint in CA capacity was identified at a MAP of 38±18 mmHg without dopamine and at 44±18, 31±14, and 24±14 mmHg with dopamine infusion rates of 10, 25, and 40 μg/kg/min (p = 0.057). Neither the index of steady-state cerebral perfusion nor cerebral venous saturation were affected by dopamine infusion.

**Conclusion:**

Dopamine infusion tended to improve CA capacity at low blood pressures while an index of steady-state cerebral blood flow and cerebral venous saturation were unaffected by dopamine infusion. Thus, dopamine does not appear to impair CA in newborn piglets.

## Introduction

Hypotension in newborn infants is associated with higher incidence of mortality and cerebral injury[1–5]. Cerebral injuries caused by hypotension are heterogeneous with both peri- and intraventricular haemorrhage as well as ischemic cerebral lesions[1–3,5]; predominantly the white matter being the most vulnerable area to hypotensive periods[6]. These cerebral injuries result in poorer long-term neurodevelopmental outcome[4,7]. Maintaining adequate perfusion of vital organs, especially the brain, is the rationale of treating hypotension in newborn infants. Dopamine raises mean arterial blood pressure (MAP) effectively[8,9] and is the most commonly used vasopressor in newborn infants[10]. It stimulates both dopaminergic and adrenergic receptors on the arterial smooth muscle cells, and has a concentration-dependent biphasic response: vasodilation at low concentrations, caused by stimulation of dopaminergic receptor, and vasoconstriction at higher concentrations due to adrenergic stimulation[11].

Hypotensive neonates treated with dopamine have poorer long-term neurodevelopmental outcome than those who have not received dopamine[1,12–14]. These observations are, however, associations and a causal relation between dopamine therapy and poorer neurodevelopmental outcome has not been established. Dopamine is not associated with greater incidence of adverse neurological outcome than other vasopressors[15].

Cerebral autoregulation (CA) is a protective mechanism that maintains a fairly stable cerebral blood flow despite fluctuations in MAP. Cerebral arteries reach their maximal compensatory vasodilation when MAP is at the lower limit of CA. Reduction in MAP below the lower limit of CA will reduce cerebral blood flow and ultimately expose the brain to ischaemia. We have previously described an association between impaired CA and dopamine infusion in very preterm infants[16], unfortunately, the study design did not allow us to determine if this relation was causal.

The observed association between poor neurodevelopmental outcome and dopamine therapy in hypotensive infants might be explained in different ways: dopamine usage might rather be an indicator of disease and vulnerability, or maybe dopamine has a direct deleterious effect on the developing brain as dopamine has been shown to cross the blood-brain-barrier in preterm infants[17]. Another concern could be if dopamine affects CA. In support of this last explanation, increased sympathetic tone induces a rightward shift of the CA curve in adult humans[18]. Due to its sympathomimetic effects, we speculate that dopamine therapy leads to a rightward shift of the CA curve. An increased cerebrovascular resistance, due to stimulation of adrenoceptors, might cause this effect. If dopamine therapy results in a rightward shift of the CA curve, infants treated with dopamine would have a higher risk of cerebral ischaemia at a MAP that is otherwise considered “safe” if the infants were not treated with dopamine. The aim of this study was to test this in a piglet model.

## Materials and methods

The Danish Animal Experiments Inspectorate approved the experimental protocol: 2014-15-0201-00123.

The sample size calculation was based on a previous study where dopamine infusion increased cerebral perfusion in piglets (gain-laser doppler flowmetry 3±1.4%/mmHg)[19]. If we assume to find the same standard deviation, we will need five piglets in each group to detect a difference in gain-laser doppler flowmetry of 3%/mmHg with 90% power and a two-sided significance level of 0.05. Therefore, we included six piglets in each dopamine infusion rate group.

### Anaesthesia and monitoring

Anaesthesia was induced by 3% isoflurane and supplemented by 5 mg/kg propofol and 20 μg/kg fentanyl and maintained on propofol 15–20 mg/kg/h and fentanyl 7 μg/kg/h. Fentanyl was discontinued after the preparation of the piglet and at least one hour before start of the protocol. A bolus of propofol or fentanyl was given if the piglet showed signs of superficial anaesthesia or pain. After tracheostomy, the piglet was connected to a pressure-controlled mechanical ventilator. Settings were adjusted to maintain PaCO_2_ between 4–6 kPa and arterial saturation above 95%. Arterial saturation and heart rate were monitored by pulse oximetry from the forepaw (Radical 7, Massimo, Hannover, Germany). Piglets were kept on a heating pad to maintain rectal temperature 38.5–39.5°C.

### Surgical preparation

The femoral arteries were cannulated with (i) an arterial line for MAP monitoring and blood gas sampling (placed in the thoracic part of aorta), and (ii) a 4 FR embolectomy-catheter with a balloon near its tip (LeMaitre Vascular, Germany) with the purpose of increasing MAP in the upper part of the body by inflating of the balloon. The arterial balloon catheter was positioned by inflating the balloon: first inflation of the balloon above the arterial line, resulting in a flat arterial blood pressure waveform. Then the arterial balloon catheter was drawn back until inflation of the balloon resulted in increased MAP along with an intact blood pressure waveform and left there. The femoral veins were cannulated with (i) a 4 FR double lumen central venous line for infusion of glucose and dopamine, and (ii) another 4 FR embolectomy-catheter with the balloon in the inferior vena cava just before the inlet into right atrium (16cm) with the purpose of decreasing cardiac preload when inflated and hereby to induce arterial hypotension. Control of the placement was done by checking that MAP was easily manipulated. In some cases, position was confirmed by x-ray.

### Cerebral monitoring

We monitored micro-vascular perfusion by laser-doppler flowmetry (LDF) (PeriFlux 5000, Stockholm, Sweden). For placement of the LDF probe in the right parietal region, a biopsy punch was used to perform a craniotomy and dura was leaved intact. The LDF probe was fixated in a specially designed metal washer glued to the skull of the piglet. In this way, we secured that measurements were from the same point on the cerebral cortex. LDF, measured as perfusion units (PU), are non-absolute values that depend on the number of moving red blood cells as well as the velocity of these cells. Therefore, relative changes in PU were used as an index of steady-state cerebral blood flow. Superior sagittal sinus was cannulised and cerebral venous blood was sampled in order to determine cerebral venous saturation. All piglets recovered for one hour after preparation.

### Randomization and protocol

**Fig 1:**
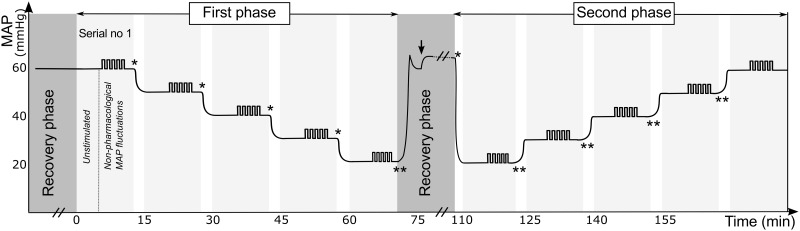
Experimental protocol. The protocol consisted of two phases: a phase where mean arterial blood pressure (MAP) was decreased and a phase where MAP was increased. At each MAP level, five small fluctuations in MAP was induced by the balloon catheter placed in aorta. We randomized the order of the two phases, whether dopamine was given in first or second phase, and the infusion rate of dopamine. In this illustration the piglet was randomized to have a decreasing MAP in the first phase and an increasing MAP in the second phase, also dopamine was randomized to be given during the second phase (arrow points to initiation of dopamine). *Indicates inflation of the venous balloon causing reduced MAP, whereas ** indicates increased MAP caused by deflation of the venous balloon. Before and between the two phases the piglet recovered for 0.5–1 hour.

The experimental protocol is illustrated in Fig 1. The protocol had two phases: a phase where MAP was decreased and a phase where MAP was increased. Hypotension was induced by inflating the balloon catheter in vena cava. We intended to decrease/increase MAP in steps of approximately 10 mmHg. We randomized the order of the two phases and whether dopamine was given during the first or second phase. Also, each piglet was randomized to receive dopamine at an infusion rate of either 10 μg/kg/min (n = 6), 25 μg/kg/min (n = 6) or 40 μg/kg/min (n = 6). These dosages were chosen based on previous findings in piglets where 15–25 μg/kg/min dopamine was required to induce a significant rise in MAP[19–21]. Therefore, the dosages represent (i) a low-dosage, that in theory would only affect the dopaminergic receptors, (ii) a blood pressure active dosage, and (iii) a supra-standard dosage.

At each level of hypotension, blood samples were drawn from the arterial line and from superior sagittal sinus. Then, to test the autoregulatory capacity, five fluctuations in MAP were induced by in- and deflating 0.4 ml saline into the aortic balloon catheter, causing rapid in- and decreases in the blood pressure, each cycle lasting 60 sec. Between the two phases (i.e MAP being either in- or decreased), the venous balloon was fully deflated while the piglet recovered. If dopamine was given during the first phase dopamine infusion rate was reduced by 10 μg/kg/min every 10 mins during the recovery phase. After dopamine infusion was stopped the piglet recovered for 30 mins. If dopamine was given in the second phase the piglet recovered for 30 mins after dopamine infusion was initiated. At the end of the protocol, piglets were euthanized by pentobarbitone (100 mg/kg) while they were still anaesthetized.

### Calculation of cerebral autoregulation capacity

CA capacity was estimated, as described by Tiecks et al[22], as a mean over five MAP fluctuations induced by in- and deflations of the arterial balloon. The calculations were based on the last 15 sec before in- and deflation of the arterial balloon; in this way, static CA was determined. Firstly, we calculated estimated cerebrovascular resistance (CVRe) as: CVRe = MAP/PU. Then CA capacity was calculated as percentage change of CVRe in relation to percentage change of MAP, i.e. CA capacity = %ΔCVRe / %ΔMAP x 100%, where %ΔCVRe = (CVRe(balloon)—CVRe(baseline)) / CVRe(baseline), and %ΔMAP = MAP(balloon)—MAP(baseline) / MAP(baseline). Thus, expression of CA capacity is a percentage of full CA capacity, i.e. perfectly working CA where MAP fluctuations result in no fluctuations in LDF.

### Statistics

The relation between MAP and the outcome variables (cerebral venous saturation, an index of steady-state cerebral blood flow as estimated by LDF, and CA capacity) were best described by a regression line with a break, i.e. two intersecting regression lines with a breakpoint: a positive slope below the breakpoint, and a horizontal line above the breakpoint. This method has previously been used to describe the relationship between MAP and cerebral blood flow[6,23]. We used non-linear regression to determine the breakpoint, i.e. the MAP where the residual sums of squares reach a minimum[23].

The following equation was used to describe relationship between one outcome variable and MAP: ‘Outcome variable’ = (MAP < breakpoint) * ((MAP—breakpoint) * slope) + plateau. The first bracket in the equation gives a dichotomous outcome– 0 when false and 1 when true. Thus, if MAP is above the determined breakpoint, the value of the first bracket is zero and the ‘outcome variable’ = plateau. On the other hand, if MAP is below the breakpoint, the value of the bracket is one and it is possible to calculate the slope of the curve below the breakpoint. We assumed ‘perfect’ autoregulation above the breakpoint, the ‘plateau’. Since each piglet only contributed five to seven data points with and without dopamine, respectively, non-linear regression fitting three parameters (breakpoint, slope, and plateau) will not converge. Therefore, we determined the common slope and plateau for all the piglets in one analysis. Thereafter we performed non-linear regression analysis for each piglet to determine individual breakpoints with the slope and plateau as fixed parameters as determined in the common analysis. These individual breakpoints were related to dopamine infusion rate by linear regression. The index of steady-state cerebral blood flow measured by LDF was logarithmic transformed to obtain variance homogeneity. We used students t-test to compare means in two groups, and paired t-test when changes in a parameter within the same piglet were tested. Values are given as mean ± standard deviation or median (range).

## Results

### Piglets

Eighteen piglets were included in the study (median age 60 hours (range 4–66 hours), weight 1.9 ± 0.2 kg). MAP before start of protocol was 59 ± 10 mmHg. Arterial saturation and arterial pCO_2_ were stable during the experiment. None of the piglets needed resuscitation during the experiment or were euthanized prior to termination of the experiment.

### Randomization

None of the outcome variables: Cerebral venous saturation, the index of steady-state cerebral blood flow, or CA capacity were affected by whether the piglets were randomized to have dopamine in the first or last phase or whether MAP went high-to-low or low-to-high.

### Dopamine and blood pressure

In the low-dosage dopamine group (10μg/kg/min) MAP was unaffected by dopamine infusion (ΔMAP 4±9 mmHg, p = 0.316), whereas MAP increased when dopamine infusion rates were 25 μg/kg/min (ΔMAP 17±8, p = 0.004) and 40 μg/kg/min (ΔMAP 23±10, p = 0.002).

### Cerebral venous saturation and dopamine

**Fig 2:**
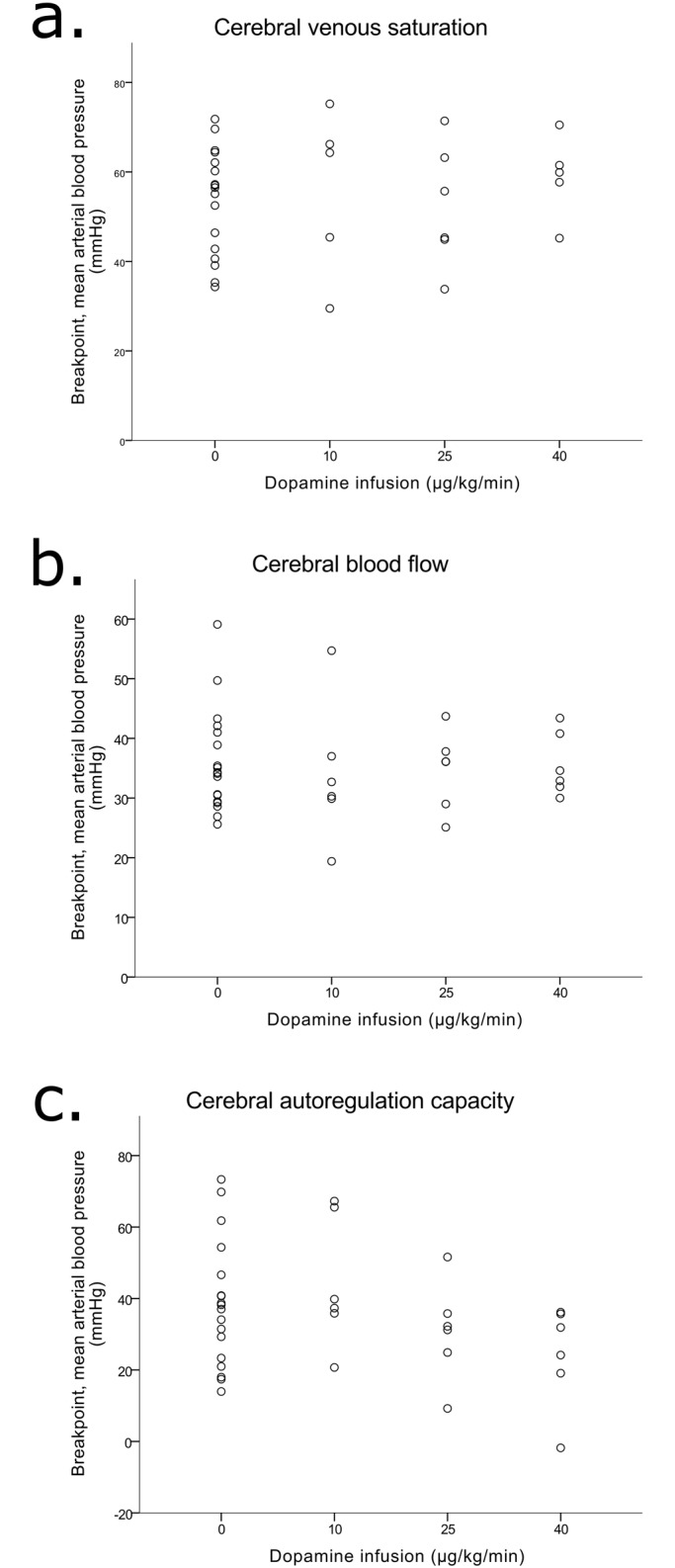
Relation between individual breakpoints of ‘outcome variables’ and dopamine infusion in 18 piglets. Each piglet was studied twice—without and with one of the three infusion rates of dopamine. (a) Cerebral venous saturation (regression coefficient = 0.09 mmHg/μg*kg^-1^*min^-1^, p = 0.558). (b) The index of steady-state cerebral blood flow (regression coefficient = -0.02 mmHg/μg*kg^-1^*min^-1^, p = 0.863). (c) CA capacity (regression coefficient = -0.36 mmHg/μg*kg^-1^*min^-1^, p = 0.057).

During the induced hypotension, MAP ranged between 14 and 82mmHg. When including all data in one non-linear regression analysis, we found a slope of 0.78%/mmHg of the cerebral venous saturation curve below the breakpoint. The plateau was placed at a cerebral venous saturation of 45%. Based on the individual determination of the breakpoints, the mean breakpoints were at 47±2 mmHg, 43±6 mmHg, 50±4 mmHg, and 53±6mmHg, without dopamine, with 10 μg/kg/min, 25 μg/kg/min, and 40 μg/kg/min, respectively. Regression coefficient of the relation between individual breakpoints of cerebral venous saturation and dopamine infusion was 0.09 mmHg/μg*kg^-1^*min^-1^ (p = 0.558) and is illustrated in Fig 2a.

### Cerebral blood flow and dopamine

The relation between the index of steady-state cerebral blood flow, as indicated by LDF, and MAP had a positive slope of 1.06%/mmHg below a breakpoint and the plateau was placed at 96% of the baseline LDF value. When the piglets did not receive dopamine the breakpoint was at 36±18 mmHg. With increasing dopamine infusion rates the breakpoints were at 30±18 mmHg, 35±10 mmHg and 38±18 mmHg. There was no trend between the individual breakpoints of an index of steady-state cerebral blood flow and dopamine infusion rate (Fig 2b) (regression coefficient of -0.02 mmHg/μg*kg^-1^*min^-1^, p = 0.863).

### Cerebral autoregulation capacity and dopamine

**Fig 3:**
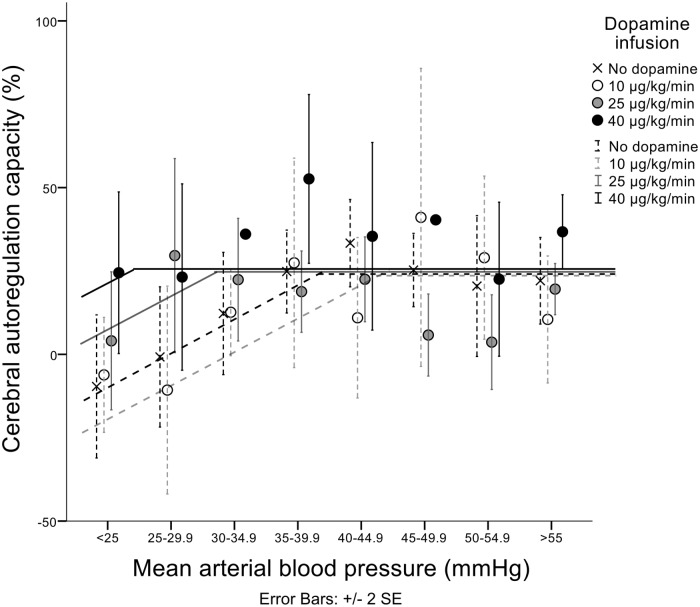
Relation between mean arterial blood pressure and cerebral autoregulation capacity. This relationship is best described by a regression line with a breakpoint and are illustrated as the non-linear regression lines in the figure. Below the breakpoint, the line had a positive slope (1.7%/mmHg); and the line is horizontal above the breakpoint. These parameters were kept fixed in the individual calculations of breakpoints. Based on individual calculations of breakpoints, the mean breakpoint without dopamine was at 38mmHg. With 10 μg/kg/min, 25 μg/kg/min and 40 μg/kg/min the mean breakpoints were at 44mmHg, 31mmHg and 24mmHg, respectively.

Like cerebral venous saturation and the index of steady-state cerebral blood flow, CA capacity had a positive slope below the breakpoint (slope 1.7%/mmHg) and the plateau was placed at a CA capacity of 26%. The breakpoint was 38±18 mmHg when the piglets did not receive dopamine. Piglets receiving dopamine infusion rates of 10 μg/kg/min, 25 μg/kg/min and 40 μg/kg/min had breakpoints at 44±18 mmHg, 31±14 mmHg and 24±14 mmHg, respectively (Fig 3). The relation between individual breakpoints of CA capacity and dopamine infusion had a regression coefficient of -0.36 mmHg/μg*kg^-1^*min^-1^, p = 0.057 (Fig 2c).

## Discussion

Our hypothesis was that dopamine affected CA negatively by inducing cerebral vasoconstriction. We used three different measures to examine our hypothesis. Cerebral venous saturation is a global estimate of the venous saturation from both hemispheres, whereas the two other measures: CA capacity, as an estimate of cerebral vasoreactivity, and the index of steady-state cerebral blood flow are based on perfusion in a very small area of cortex as interrogated by the laser Doppler light probe. Surprisingly, CA capacity rather tended to improve with increasing infusion rates of dopamine while there was no suggestion of an effect of dopamine treatment on the index of steady-state cerebral blood flow or cerebral venous saturation at low levels of MAP.

### Cerebral venous saturation and dopamine

CA maintains cerebral blood flow by cerebral vasodilation when MAP is low. Below the lower limit of CA, cerebral blood flow falls in proportion with the fall in MAP. As cerebral metabolic rate of oxygen is the product of cerebral blood flow and cerebral oxygen extraction and if cerebral metabolic rate of oxygen is constant, then changes in cerebral oxygen extraction reflect changes in cerebral blood flow [24]. We chose to use changes in cerebral venous saturation as a simple indicator of the failure of CA to maintain sufficient cerebral blood flow during hypotension. Althought oxygen extraction, and consequently cerebral venous saturation, is influenced by changes in haemoglobin and haemoglobin did decrease over time (first measure of haemoglobin 7.5±1.7 vs. last measure of haemoglobin 6.5±1.3), the balanced study design should have eliminated this effect.

Superior sagittal sinus blood comes from both hemispheres but is dominated by blood from cortex with higher blood flow. White matter is more vulnerable to ischemia during hypotension [6], thus we may potentially have underestimated white matter ischemia.

The MAP breakpoints, i.e. where the cerebral venous saturation began to drop in response to a decrease in MAP, were unaffected by dopamine infusion (Fig 2a). This suggests that dopamine did not affect global cerebral blood flow negatively. Tsuji et al, however, showed that oxygen extraction was much less affected at low MAP than was blood flow in newborn piglets[25], presumably due to a decrease in oxygen consumption. Therefore, it is possible that cerebral venous saturation was a relatively insensitive indicator of the failure of autoregulation, but such insensitivity need not interfere with the effect of dopamine.

### Cerebral blood flow and dopamine

We estimated an index of steady-state cerebral blood flow at each level of hypotension in parietal cortex by LDF. Dopamine infusion did not affect the location of breakpoints (Fig 2b). If dopamine infusion had a negative effect on CA, we would expect a rightward shift of the ‘MAP-cerebral blood flow’ curve, caused by cerebral vasoconstriction.

### Cerebral autoregulation capacity and dopamine

CA capacity quantifies the cerebral arteries’ ability to adjust cerebrovascular resistance in response to induced fluctuations in MAP, i.e. the vasoreactivity. We were surprised that CA capacity rather tended to improve at low MAP with higher infusion rates of dopamine (Figs 2c and 3). A theoretical explanation might be that dopamine infusion induces cerebral vasodilation—and not cerebral vasoconstriction. Dopamine does induce vasodilation at low concentrations and vasoconstriction at higher concentrations. In isolated human pial arteries vasodilation has been observed in the concentration range 5 x 10^−8^ M to 2 x 10^−5^ M[26]. If we assume, that dopamine penetrates the blood-brain-barrier in a passive manner in newborn piglets, and when extrapolating from the proportion of dopamine that penetrated the blood-brain-barrier in preterm infants[17], our infusion rates of dopamine would lead to a concentration of dopamine in the cerebrospinal fluid in the order of 10^−7^–10^−6^ M. Therefore, dopamine in the infusion rates that we used could, in theory, induce vasodilation. However, these theoretical considerations are not supported by the two other outcomes in this study. Another explanation could be that dopamine improved vasoreactivity and therefore reduced the degree of cerebral vasoparalysis at low MAP. In line with this explanation, dopamine therapy has been found to prevent loss of CA during hypotension in simulated traumatic brain injury; and restore the ability of pial arteries to vasodilate in response to hypotension[27]. Also, it has been shown that infusion of dopamine in high dosages (20–30 μg/kg/min) in 10 days old piglets restored CA, eventhough the piglets were anaesthetized with isoflurane[28]. We consider it most prudent to conclude that dopamine treatment does not appear to affect cerebral autoregulation negatively in the neonatal piglet.

### Strengths and limitations

The advantage of our study is that we compared cerebrovenous oxygenation, an index of steady-state cerebral blood flow, and CA capacity at comparable levels of MAP with and without dopamine infusion. This is important because an increase in MAP, caused by dopamine infusion, in itself might affect these variables.

Our study also has limitations. First, we used healthy newborns piglets to mimic hypotensive newborn infants. We do not know if the presence and development of dopaminergic and adrenergic receptors are identical in piglets and infants. The piglets were healthy, in contrast, most infants who are treated with dopamine are distressed and might have increased basal sympathetic tone. Second, we used MAP instead of cerebral perfusion pressure in our description of CA. Minor changes from normal intracranial pressure to lower intracranial pressure might be expected when we induced hypotension. However, we would not expect intracranial pressure to vary significantly in our experimental design and Brady et al showed that in gradually hypotensive newborn piglets an estimate of CA using MAP was highly comparable to an estimate of CA using cerebral perfusion pressure[29]. Third, an index of steady-state cerebral blood flow was estimated by LDF. LDF measures perfusion within a very small area of cortex. It would have been valuable to determine the global cerebral blood flow in absolute terms by using the colored microsphere technique. However, colored microspheres only allow a limited number of measurements in each animal and our main interest was to monitor changes in cerebral blood flow continuously to estimate CA. This change in CBF cannot be determined by microsphere technique. It has been shown that LDF correlates well with several gold standard methods for measuring cerebral blood flow, including the microsphere technique, but also that relative changes in LDF may underestimate the relative change in CBF by 10–20%[30,31]. In line with these observations, LDF has been shown to be a reliable method to determine the lower limit of CA[32,33], which was the purpose of our study. LDF measures were calculated as changes in PU in each level of hypotension relative to the baseline value. We did not adjust for the flux at zero perfusion. In our pilot-piglets and the first included piglets (n = 6) the flux after euthanization was negligible (PU<5) so we decided to ignore it.

When we calculated the breakpoints in the non-linear regression analysis we assumed that the slope below the breakpoint was the same without and in all three dosages of dopamine. It is possible, that the slope is affected by dopamine dosage; but the study was not powered to study this.

Another concern might be if our anaesthetic procedure affects our measurements. Piglets were initially sedated by isoflurane. It is well described that isoflurane impairs CA[34] and therefore, isoflurane was stopped as soon as an iv-line was established. Propofol was chosen as the anaesthetic agent during the experiment because it has been shown to have no effect on CA[35]. In our protocol, propofol and fentanyl could be supplemented by bolus injections if the piglet showed signs of superficial anaesthesia or pain. However, bolus injections were only administered in the preparation phase. Therefore, we find it unlikely that these bolus injections could influence cerebral blood flow or cerebrovascular resistance during the experimental protocol and bias our findings.

To ensure equal distribution of design-induced bias, data from each piglet was obtained with and without dopamine, and with MAP both being lowered and raised. Furthermore, to exclude a time-dependent bias, the order of dopamine/not dopamine and increasing/decreasing MAP was randomized between piglets. Ideally, each piglet should have been studied without dopamine and with all three infusion rates of dopamine to eliminate the effect of variability among piglets. We did not attempt this study design because we expected, based on previous experience with experiments on newborn piglets, deterioration of the piglet model over four periods with hypotension and a long experimental time. We considered that measurements during two periods with severe hypotension were a more robust design. Actually, severe hypotension might induce cerebral ischemia and this could affect autoregulation afterwards. Despite severe hypotension lasting approximately 10 min, we did not observe any cardiac arrest. Neither did we see any influence on our measurements (no statistical significant differences between dopamine-first and dopamine-last data). In this model the short duration of hypotension and decreased blood flow did not lead to impairment of CA. In an experimental setting with hypotensive adult cats, ischemic brain lesions were only seen when MAP was lowered to 50% of the lower limit of CA for at least 15 min[36].

## Conclusion

Dopamine tended to improve cerebral autoregulation capacity at low arterial blood pressure; however, a beneficial effect of dopamine was not confirmed by improved cerebral blood flow or cerebrovenous oxygen saturation. We conclude that dopamine therapy does not appear to affect cerebral autoregulation negatively in hypotensive newborn piglets.

## Supporting information

S1 File Data file. (SAV)

S2 File C3Rs ARRIVE guidelines checklist. (PDF)

## References

[1] Fanaroff JM, Wilson-Costello DE, Newman NS, Montpetite MM, Fanaroff AA. Treated hypotension is associated with neonatal morbidity and hearing loss in extremely low birth weight infants. Pediatrics. 2006 4;117(4):1131–5. 10.1542/peds.2005-1230 16585307

[2] Meek JH, Tyszczuk L, Elwell CE, Wyatt JS. Low cerebral blood flow is a risk factor for severe intraventricular haemorrhage. Arch Dis Child Fetal Neonatal Ed. 1999 7;81:F15–8. 10375356 10.1136/fn.81.1.f15 PMC1720962

[3] Al Tawil KI, El Mahdy HS, Al Rifai MT, Tamim HM, Ahmed IA, Al Saif SA. Risk factors for isolated periventricular leukomalacia. Pediatr Neurol. Elsevier Inc.; 2012 3;46(3):149–53. 10.1016/j.pediatrneurol.2011.12.008 22353288

[4] Low J, Froese A, Galbraith R, Smith J, Sauerbrei E, Derrick E. The association between preterm newborn hypotension and hypoxemia and outcome during the first year. Acta Paediatr. 1993;82:433–7. 7686060 10.1111/j.1651-2227.1993.tb12717.x

[5] Miall-Allen VM, de Vries LS, Whitelaw AGL. Mean arterial blood pressure and neonatal cerebral lesions. Arch Dis Child. 1987;62(10):1068–9. 3314723 10.1136/adc.62.10.1068 PMC1778679

[6] Børch K, Lou HC, Greisen G. Cerebral white matter blood flow and arterial blood pressure in preterm infants. Acta Paediatr. 2010;99:1489–92. 10.1111/j.1651-2227.2010.01856.x 20456278 PMC3068289

[7] Fanaroff AA, Fanaroff JM. Short- and long-term consequences of hypotension in ELBW infants. Semin Perinatol. 2006 6;30(3):151–5. 10.1053/j.semperi.2006.04.006 16813974

[8] Pellicer A, Valverde E, Elorza MD, Madero R, Gayá F, Quero J, et al Cardiovascular support for low birth weight infants and cerebral hemodynamics: a randomized, blinded, clinical trial. Pediatrics. 2005 6;115(6):1501–12. 10.1542/peds.2004-1396 15930210

[9] Zhang J, Penny DJ, Kim NS, Yu VY, Smolich JJ. Mechanisms of blood pressure increase induced by dopamine in hypotensive preterm neonates. Arch Dis Child Fetal Neonatal Ed. 1999 9;81(2):F99–104. 10448176 10.1136/fn.81.2.f99 PMC1720986

[10] Rios DR, Moffett BS, Kaiser JR. Trends in pharmacotherapy for neonatal hypotension. J Pediatr. Elsevier Inc; 2014 7 16;165:697–701. 10.1016/j.jpeds.2014.06.009 25039051

[11] Amenta F, Barili P, Bronzetti E, Felici L, Mignini F, Ricci A. Localization of dopamine receptor subtypes in systemic arteries. 2000;22(3):277–88. 10.1081/ceh-100100077 10803733

[12] Barrington KJ. Hypotension and shock in the preterm infant. Semin Fetal Neonatal Med. 2008 2;13(1):16–23. 10.1016/j.siny.2007.09.002 17974512

[13] Dempsey EM, Al Hazzani F, Barrington KJ. Permissive hypotension in the extremely low birthweight infant with signs of good perfusion. Arch Dis Child Fetal Neonatal Ed. 2009 7;94(4):F241–4. 10.1136/adc.2007.124263 19174413

[14] Batton B, Li L, Newman NS, Das A, Watterberg KL, Yoder BA, et al Early blood pressure, antihypotensive therapy and outcomes at 18–22 months’ corrected age in extremely preterm infants. Arch Dis Child—Fetal Neonatal Ed. 2016;101:F201–6. 10.1136/archdischild-2015-308899 26567120 PMC4849123

[15] Sassano-Higgins S, Friedlich P, Seri I. A meta-analysis of dopamine use in hypotensive preterm infants: blood pressure and cerebral hemodynamics. J Perinatol. 2011 10;31(10):647–55. 10.1038/jp.2011.2 21273985

[16] Eriksen VR, Hahn GH, Greisen G. Dopamine therapy is associated with impaired cerebral autoregulation in preterm infants. Acta Paediatr. 2014;103:1221–6. 10.1111/apa.12817 25266994

[17] Seri I, Tulassay T, Kiszel J, Sulyok E, Ertl T, Bódis J, et al Effect of low-dose dopamine therapy on catecholamine values in cerebrospinal fluid in preterm neonates. J Pediatr. 1984 9;105(3):489–91. 6470873 10.1016/s0022-3476(84)80035-9

[18] Paulson OB, Strandgaard S, Edvinsson L. Cerebral autoregulation. Cerebrovasc Brain Metab Rev. 1990;2(2):161–92. 2201348

[19] Hahn GH, Heiring C, Pryds O, Greisen G. Cerebral vascular effects of hypovolemia and dopamine infusions: a study in newborn piglets. Acta Paediatr. 2012 7;101(7):736–42. 10.1111/j.1651-2227.2012.02666.x 22404282

[20] Ferrara JJ, Dyess DL, Peeples GL, Christenberry DP, Roberts WS, Tacchi EJ, et al Effects of dopamine and dobutamine on regional blood flow distribution in the neonatal piglet. Ann Surg. 1995 5;221(5):531–40. 7748035 10.1097/00000658-199505000-00011 PMC1234634

[21] Hahn GH, Hyttel-Sorensen S, Petersen SM, Pryds O, Greisen G. Cerebral effects of commonly used vasopressor-inotropes: a study in newborn piglets. PLoS One. 2013 1;8(5):e63069 10.1371/journal.pone.0063069 23700412 PMC3659109

[22] Tiecks FP, Lam AM, Aaslid R, Newell DW. Comparison of static and dynamic cerebral autoregulation measurements. Stroke. 1995;26:1014–9. 7762016 10.1161/01.str.26.6.1014

[23] Munro MJ, Walker AM, Barfield CP. Hypotensive extremely low birth weight infants have reduced cerebral blood flow. Pediatrics. 2004 12;114(6):1591–6. 10.1542/peds.2004-1073 15574619

[24] Kissack CM, Garr R, Wardle SP, Weindling AM. Cerebral fractional oxygen extraction is inversely correlated with oxygen delivery in the sick, newborn, preterm infant. J Cereb Blood Flow Metab. 2005;25:545–53. 10.1038/sj.jcbfm.9600046 15744253

[25] Tsuji M, DuPlessis A, Taylor G, Crocker R, Volpe JJ. Near infrared spectroscopy detects cerebral ischemia during hypotension in piglets. Pediatr Res. 1998;44:591–5. 10.1203/00006450-199810000-00020 9773851

[26] Toda N. Dopamine vasodilates human cerebral artery. Experientia. 1983;39(10):1131–2. 6311609 10.1007/BF01943144

[27] Armstead WM, Riley J, Vavilala MS. Dopamine prevents impairment of autoregulation after traumatic brain injury in the newborn pig through inhibition of up-regulation of endothelin-1 and extracellular signal-regulated kinase mitogen-activated protein kinase. Pediatr Crit Care Med. 2013 2;14(2):e103–11. 10.1097/PCC.0b013e3182712b44 23314184 PMC3567252

[28] Nachar R a, Booth E a, Friedlich P, Borzage M, Soleymani S, Wider MD, et al Dose-dependent hemodynamic and metabolic effects of vasoactive medications in normotensive, anesthetized neonatal piglets. Pediatr Res. 2011 11;70(5):473–9. 10.1203/PDR.0b013e31822e178e 21775923

[29] Brady KM, Mytar JO, Kibler KK, Hogue CW, Lee JK, Czosnyka M, et al Noninvasive autoregulation monitoring with and without intracranial pressure in the naive piglet brain. Anesth Analg. 2010 7;111(1):191–5. 20519421 10.1213/ANE.0b013e3181e054ba PMC5505736

[30] Müller T, Löhle M, Schubert H, Bauer R, Wicher C, Antonow-Schlorke I, et al Developmental changes in cerebral autoregulatory capacity in the fetal sheep parietal cortex. J Physiol. 2002;539(Pt 3):957–67. 10.1113/jphysiol.2001.012590 11897864 PMC2290182

[31] Bishai JM, Blood AB, Hunter CJ, Longo LD, Power GG. Fetal lamb cerebral blood flow (CBF) and oxygen tensions during hypoxia: a comparison of laser Doppler and microsphere measurements of CBF. J Physiol. 2003;546:869–78. 10.1113/jphysiol.2002.025270 12563011 PMC2342576

[32] Tonnesen J, Pryds A, Larsen EH, Paulson OB, Hauerberg J, Knudsen GM. Laser Doppler flowmetry is valid for measurement of cerebral blood flow autoregulation lower limit in rats. Exp Physiol. 2005;90(3):349–55. 10.1113/expphysiol.2004.029512 15653714

[33] Brady KM, Lee JK, Kibler KK, Easley RB, Koehler RC, Shaffner DH. Continuous measurement of autoregulation by spontaneous fluctuations in cerebral perfusion pressure: comparison of 3 methods. Stroke. 2008 9;39(9):2531–7. 10.1161/STROKEAHA.108.514877 18669896 PMC2566962

[34] Bruins B, Kilbaugh TJ, Margulies SS, Friess SH. The anesthetic effects on vasopressor modulation of cerebral blood flow in an immature swine model. Anesth Analg. 2013;116(4):838–44. 10.1213/ANE.0b013e3182860fe7 23460561 PMC3606687

[35] Strebel S, Lam A, Matta B, Mayberg T, Aaslid R, Newell D. Dynamic and static cerebral autoregulation during isoflurane, desflurane, and propofol anesthesia. Anesthesiology. 1995;83:66–76. 7605020 10.1097/00000542-199507000-00008

[36] Graham DI, Fitch W, MacKenzie ET, Harper AM. Effects of hemorrhagic hypotension on the cerebral circulation. III. Neuropathology. Stroke. 1979;10(6):724–7. 524414 10.1161/01.str.10.6.724

